# Robot touch with speech boosts positive emotions

**DOI:** 10.1038/s41598-022-10503-6

**Published:** 2022-04-27

**Authors:** Taishi Sawabe, Suguru Honda, Wataru Sato, Tomoki Ishikura, Masayuki Kanbara, Sakiko Yoshikawa, Yuichiro Fujimoto, Hirokazu Kato

**Affiliations:** 1 Division of Information Science, Nara Institute of Science and Technology (NAIST), 8916-5 Takayama, Ikoma, Nara 630-0192 Japan; 2 Psychological Process Research Team, Guardian Robot Project, RIKEN, 2-2-2 Hikaridai, Seika, Soraku District, Kyoto, 619-0288 Japan; 3 Kyoto University of the Arts, 2-116 Uryuyama Kitashirakawa, Sakyo, Kyoto, Kyoto 606-8271 Japan

**Keywords:** Physiology, Psychology

## Abstract

A gentle touch is an essential part of human interaction that produces a positive care effect. Previously, robotics studies have shown that robots can reproduce a gentle touch that elicits similar, positive emotional responses in humans. However, whether the positive emotional effects of a robot’s touch combined with speech can be enhanced using a multimodal approach remains unclear. This study supports the hypothesis that a multimodal interaction combining gentle touch and speech by a robot enhances positive emotional responses. Here, we conducted an experiment using a robotic arm to perform a gentle touch combined with speech and compared three conditions: touch alone, speech alone, and touch with speech. We assessed participants’ subjective ratings of valence, arousal, and human likeliness using subjective emotional responses. Furthermore, we recorded facial electromyography (EMG) from the corrugator supercilii and zygomaticus major muscles and measured skin conductance levels (SCLs) as physiological emotional responses. Our results show that touch combined with speech elicited higher subjective valence and arousal ratings, stronger zygomaticus major EMG and SCL activities than touch alone. The results suggest that the positive emotional effects of robotic touch can be boosted by combining elements of speech.

## Introduction

Touch plays an essential role in communication between people^1^. The social touch, occurring between individuals^2^, is used to ease interpersonal communication and express personal feelings for other people^3^. We intuitively perform acts of gentle touch daily, for example, when we care for or reassure a sick or anxious person^4,5^. Some well-known caring practices, such as Humanituide and Taktil massage, use gentle touch to improve emotional well-being in the nursing and medical fields^6–10^. Its positive effects are supported by empirical evidence, including several clinical psychological and psychophysiological studies showing that touch has a facilitative effect on people’s health^11–14^. Some experimental psychophysiological studies have shown that touch at a particular speed (i.e., stimulating C-tactile afferent at 3-10 cm/s) elicited subjective and physiological positive emotional responses, such as heightened valence and zygomatic major EMG activity^15–19^.

Presently, there are barriers to providing sufficient touch to every patient who might benefit from it. The increasing number of single-elderly persons living alone, and a shortage of healthcare workers, such as nurses^20,21^. In response to these factors, recent robotics studies have attempted to develop touch care robots. These studies suggest that touch interactions performed by robots offer a suitable substitute, producing positive experiences for human patients^22,23^. Another study showed that gentle touch including stroke motion from robots also elicits similar positive emotional responses automatically^24,25^. These findings also found that while a gentle touch by a robot can produce positive emotional responses for humans, a robot touch without adequate communication or consent can be experienced as violent^26^.

However, whether the positive emotional effects of a robot’s touch combined with speech can be enhanced using a multimodal approach remains unclear. Although no robotics studies have focused on the subject, several psychological field studies have shown that the most effective touch care methods incorporate simultaneous touch and calm, caring talk^27–29^. Additionally, some previous robotics studies produced empirical evidence^30–32^ and theoretically proposed^33–35^ that a robot’s gentle touch combined

with speech can effectively induce positive emotions in humans. However, these studies did not systematically compare the differences between touch alone and touch with speech. Therefore, whether the positive emotional effects of a robot’s touch could be enhanced using a multimodal approach combined with speech remains unproven. Based on these data, we hypothesized that a multimodal interaction combining touch and sound boosts positive emotional responses of touch by robots.

**Figure 1:**
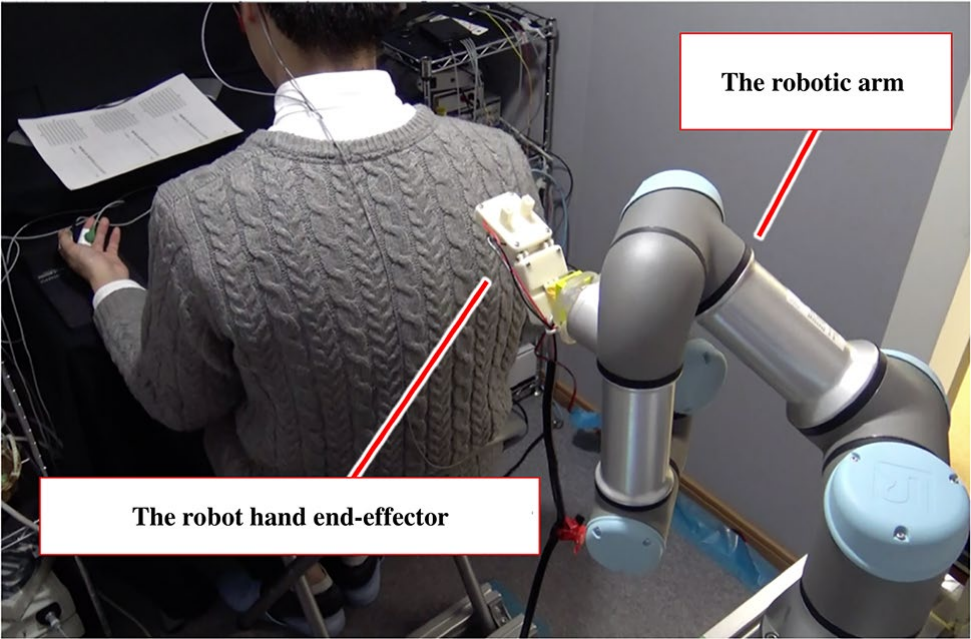
The system architecture of speech-enabled gentle touch robot using a robotic arm.

This study aimed to demonstrate a robot that combines touch and speech can more effectively generate positive emotional responses in humans. Figure 1 shows the system architecture of the gentle touch robot which consists of a robotic arm with a hand end-effector to perform a gentle touch on the participant’s upper back at the speed required to stimulate C-tactile afferents. The robot was equipped with a speaker to reproduce speech, the contents of which were sampled from a speech in nursing care situations. We compared three possible conditions: touch alone, speech alone, and touch with speech. To measure subjective emotional responses, participants were asked to rate their own valence and arousal during the stimulus presentation. For touch and speech, we tested only one touch and one speech type. For subjective ratings, we also assessed the rating of human likeliness to test the hypothesis that multimodal communication could increase the human likeliness of robots^38^. For the objective measure of emotional responses, which can offset subjective bias in ratings^36^, facial electromyography (EMG) of the corrugator supercilii and zygomaticus major muscles and skin conductance level (SCL) activities were recorded, which were known to be linked to subjective emotional valence and arousal responses, respectively^37^. We predicted that touch with speech would result in higher subjective valence and arousal ratings, and simultaneously have a greater effect on the correspondent physiological response patterns (i.e., lower corrugator supercilii EMG activity, higher zygomaticus major EMG activity, and higher SCL activity) than touch alone.

## Results

This study recorded 35 participants. However, we only analyzed the dataset of 31 participants due to technical problems.

### Subjective ratings

**Figure 2:**
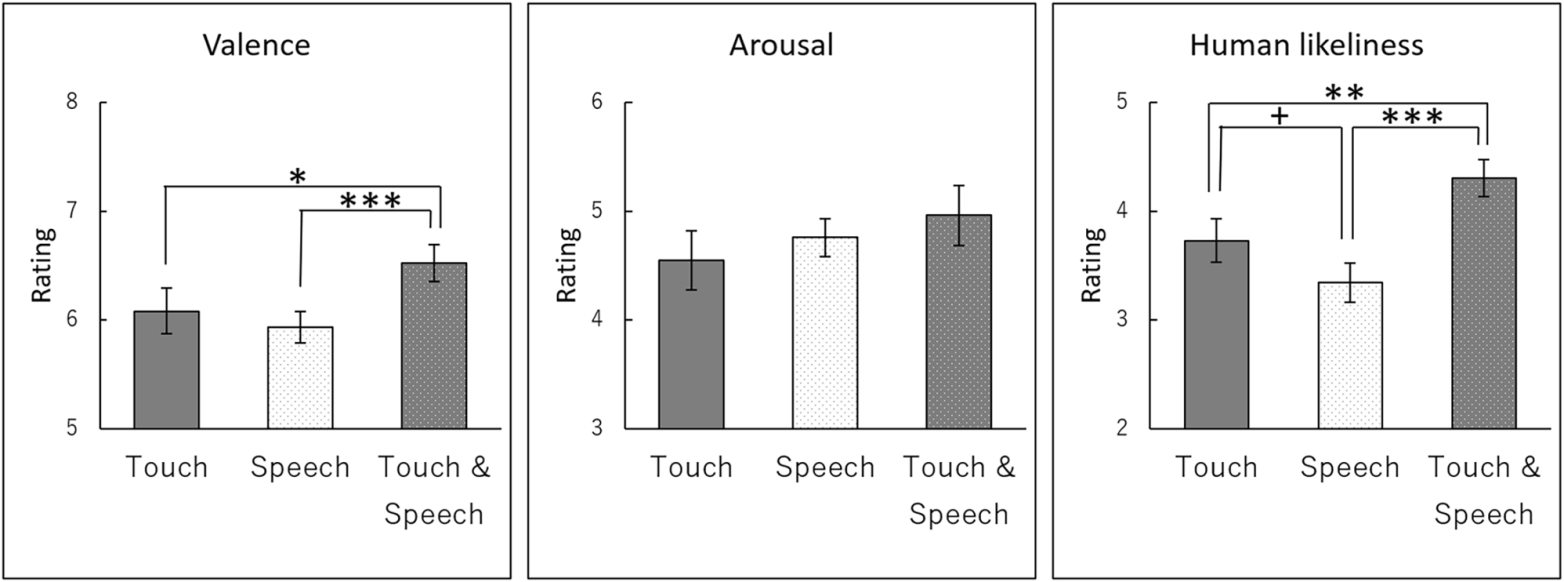
Mean (with standard error) subjective evaluations of valence, arousal, and human likeliness. ***, *p* < 0.001; **, *p* < 0.01; *, *p* < 0.05.

Figure 2 shows the results of the subjective ratings of valence, arousal, and human likeliness. First, we tested the global effect of condition on subjective ratings (i.e., valence, arousal, and human likeliness) using a repeated-measure multivariate analysis of variance (MANOVA). Added to the factor of conditions (touch alone, speech alone, and touch with speech), a block factor (first, second) was added to exploratorily test the effect of presentation order (e.g., learning). The results of the two-way MANOVA showed a significant effect on the condition, *F*(6,25) = 6.49, *p* < 0.001, *η*^2^_*p*_ = 0.609. The block factor had no significant effect, *F*(3,28) = 2.27, *p* = 0.102, *η*^2^_*p*_ = 0.196, or condition × block interaction, *F*(6,25) = 0.93, *p* = 0.491, *η*^2^_*p*_ = 0.182).

We further evaluated the effect of condition on each of subjective ratings using univariate analyses of variance (ANOVAs) with a factor of condition and follow-up multiple comparisons using Ryan method. For the valence ratings, the main effect of condition was significant, *F*(2,60) = 6.51, *p* = 0.003, *η*^2^_*p*_ = 0.178. Multiple comparisons showed that the valence ratings for touch with speech (*M* = 6.52, *SD* = 0.95) were significantly more positive than speech alone (*M* = 5.94, *SD* = 0.82), *t*(60) = 3.47, *p* = 0.001, 95%CI [0.17, 1.01], and touch alone (*M* = 6.08, *SD* = 1.14), *t*(60) = 2.60, *p* = 0.012, 95%CI [0.05, 0.83].

The analysis on the arousal ratings did not show significant main effect of condition, *F*(2,60) = 1.21, *p* = 0.305, *η*^2^_*p*_ = 0.039. A significant main effect of condition was found for the human likeliness ratings, *F*(2,60) = 12.38, *p* < 0.001, *η*^2^_*p*_ = 0.292. Multiple comparisons showed that the human likeliness ratings for touch with speech (*M* = 4.30, *SD* = 0.92) were evaluated as significantly more humanlike than speech alone (*M* = 3.34, *SD* = 0.97), *t*(60) = 4.94, *p* < 0.001, 95%CI [0.48, 1.44], and touch alone (*M* = 3.73, *SD* = 1.09), *t*(60) = 2.97, *p* = 0.004, 95%CI [0.13, 1.02]. The human likeliness ratings also showed a non-significant tendency that touch alone was higher than speech alone, *t*(60) = 1.98, *p* = 0.053, 95%CI [0.00, 0.77].

### Physiological activity

**Figure 3:**
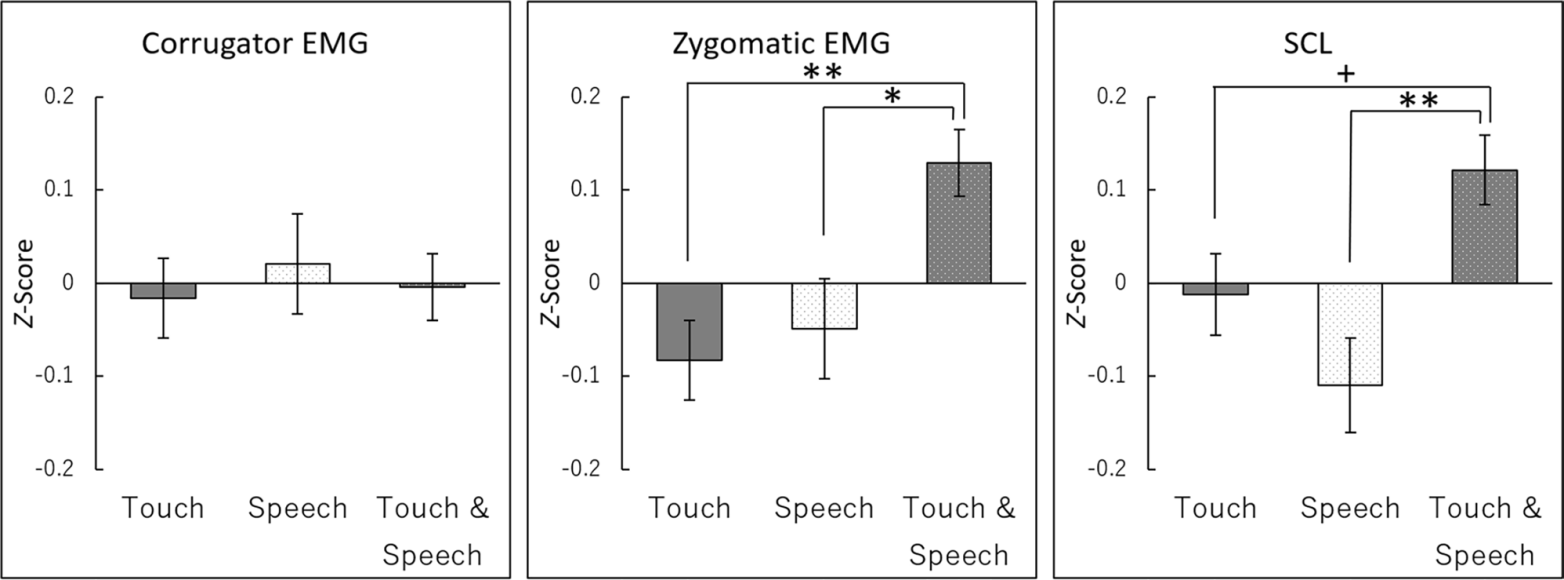
Mean (with standard error) standardized scores of facial electromyography (EMG) from the corrugator supercilii and zygomatic major muscles and skin conductance level (SCL). **, *p* < 0.01; *, *p* < 0.05; + , *p* < 0.10.

To measure the physiological activity, physiological data were standardized for each participant, as shown in Fig. 3. We analyzed the physiological data similar to the subjective ratings. A repeated-measures MANOVA for physiological measures (i.e., corrugator supercilii EMG, zygomatic major EMG, and SCL) with conditions and blocks showed only a significant effect, *F*(6,25) = 3.83, *p* = 0.008, *η*^2^_*p*_ = 0.479. No other significant effects or interactions were recorded (main effect of block: *F*(3,28) = 1.57, *p* = 0.220, *η*^2^_*p*_ = 0.144; condition × block interaction: *F*(6,25) = 0.72, *p* = 0.641, *η*^2^_*p*_ = 0.147).

The follow-up ANOVA with the corrugator supercilia EMG condition as a factor did not show any significant effect since, *F*(2,60) = 0.10, *p* = 0.901, *η*^2^_*p*_ = 0.003. The zygomatic major EMG recorded a significant effect, *F*(2,60) = 4.30, *p* = 0.018, *η*^2^_*p*_ = 0.125. Multiple comparisons showed that the zygomatic major EMG activity was significantly higher for touch with speech (*M* = 0.13, *SD* = 0.20) than for touch alone (*M* = −0.08, *SD* = 0.23), *t*(62) = 2.73, *p* = 0.008, 95%CI [0.02, 0.40], and speech alone (*M* = −0.05, *SD* = 0.29), *t*(60) = 2.29, *p* = 0.025, 95%CI [0.00, 0.35]. For the SCL, there was a significant main effect of condition, *F*(2,60) = 4.57, *p* = 0.014, *η*^2^_*p*_ = 0.132. Multiple comparisons showed that SCL was significantly higher for touch with speech (*M* = 0.12, *SD* = 0.21) than for speech alone (*M* = −0.11, *SD* = 0.28), *t*(60) = 3.01, *p* = 0.004, 95%CI [0.04, 0.42]. Moreover, there was a nonsignificant tendency of higher SCL activity for touch with speech than for touch alone (*M* = −0.01, *SD* = 0.24), *t*(60) = 1.73, *p* = 0.090, 95%CI [−0.08, 0.28].

## Discussion

The results of the subjective ratings showed that the touch and speech performed by the robotic arm induced higher valence ratings than touch alone. From these subjective ratings, physiological data showed that touch with speech elicited stronger zygomatic major EMG activity than touch alone. Furthermore, the results of subjective valence ratings showed that the “touch only” condition induced positive emotional states (>6) compared with the neutral state (i.e., 5), suggesting that robot touch induced positive emotional states as reported with human touch^20^. Nevertheless, no robotics study to date has investigated the multimodal effects of touch and speech. The existence of such multimodal interaction has been investigated in psychological studies on human touch^27–29^, although no previous studies have looked at the effectiveness of touch and speech on subjective and physiological emotional responses. Pushing the boundaries of and blurring the line between robotics and psychological studies, our study provides the first evidence that multimodal touch and speech interactions by robots can induce heightened positive emotional responses than touch alone.

The results of human likeliness ratings showed that a combination of touch and speech resulted in higher human likeliness ratings than interactions involving either touch alone or speech alone. These data agree with the previous proposal that multimodal communication increased the human likeliness of robots, despite the researchers testing the effect of speech with gestures not touch with speech^38^. Our data analysis shows that multimodal touch can enhance positive emotional responses and the human likeliness of robot touch.

Our findings have practical applications in addressing the lack of touch for patients in nursing or medical fields and even as a daily life treatment. If people are given more opportunities to receive touch care from robots in their daily, it will to a healthier lifestyle both physically and mentally.

Several limitations of this study should be acknowledged. First, we used the within-subjects design, and hence, one condition may have influenced another (e.g., learning). Although we counterbalanced the order of conditions and recorded no evident effect of block in MANOVAs, further studies using the between-subject design are needed to show the facilitative effect of robot multimodal touch more clearly. Second, participants’ personality traits were not examined. As a previous study has reported that there are reliable individual tendencies in comfort with receiving human touch^39^, such tendencies can modulate the effect of the robot multimodal touch, thereby presenting an essential focus for future research. Likewise, we did not control participants’ clothes, and hence, the possibility of varying touch effects across individuals exists depending on their clothes. This factor should be controlled to reduce individual differences. Third, we tested only the subjective ratings of valence and arousal. Debates on the representation of these measures and the relationship between them persist. Furthermore, the investigation of other ratings would be needed to clarify the subjective effect of robots’ multimodal touch. Fourth, we investigated only the global scale of the subjective ratings and averaged physiological data. Because a previous study has measured continuous subjective ratings and analyzed the temporal dynamics of physiological changes^37^, investigating dynamic patterns of emotional responses may deepen the effect of robot multimodal touch. Finally, we tested only one touch and speech type, respectively thereby restricting the generalizability of our findings. Furthermore, our speech contained heterogeneous contents of which some are interpreted as questioning without requesting replying, and may have influenced subjective and/or physiological reactions. Future studies must investigate other touch and speech types to test the reliability of the facilitative effect of robot touch with speech.

## Methods

### Participants

We analyzed the data of 31 Japanese volunteers (12 females; M ± SD age, 22.8 ± 3.9 years). We determined the sample size using an a priori power analysis. We used G*Power software^40^ (ver. 3.1.9.2) and assumed to conduct a one-way repeated-measures ANOVA with a factor of three levels with an *α* level of 0.05, power of 0.80, correlation of 0.5, *ε* of 1, and effect size *f* of 0.25 (medium). The results indicated that 28 participants would be required. Although additional 4 volunteers participated, their data were not analyzed due to equipment failure. The informed consent was obtained from all volunteers and/or their legal guardians. This study was approved by the Ethics Committee of Nara Institute of Science and Technology and was conducted according to institutional ethical provisions and the Declaration of Helsinki.

### Experimental system architecture

This study required a robotic arm system to perform gentle touch and mimic speech at the same time. As an instrument of gentle touch, a robotic arm UR3 made by Universal Robots^41^ was used for the arm parts, and hand parts, and a single-degree-of-freedom end-effector with a spring mechanism attached to the robotic arm was used, referring to the experiment by Toyoshima^25^. The end-effector consisted of a ni-chrome wire and a temperature sensor that could control the temperature of the end-effector itself. The robot’s gentle touch action was performed by specifying the starting point, stroking area, stroking force, and stroking speed, all based on the results of preliminary experiments, in which small groups (*n* < 10) different from those in the main experiment were tested using the same system. When the gentle touch motion was initiated, the robotic arm approached the starting point and made contact with the participant’s back on the cloth to perform a gentle touch. After the initial contact, the robotic arm applied pressure to the participant’s back up to the predetermined value of the force sensors. Then, by moving at a predetermined speed in the gentle touch area, while applying the specified amount of pressure to the participant’s back, the robot performed a gentle touch. During the gentle touch motion from the top to the bottom of the participant’s back in the specific distance, acceleration and deceleration occurred near the start point and endpoint performed, which simulated a natural human touch. The number of touch strokes was based on the mora/s of speech. As for the mechanism of speech, a plugin speaker was used to reproduce speech by playing a text to voice file created by using a speech synthesis system called VoiceText Web API^42^. Speech synthesis was performed at the specified speed by converting the speed of speech (mora/s) to VoiceText Web API’s original parameters, and by using the formula determined by Nakamura^43^. Using a speech synthesis system to generate speech could exclude the effect of speech quality (pitch, volume, and speed). The speed of speech was determined uniquely within the range of everyday Japanese speech cadence and the “speech pause” was adjusted so that the turn in the stroking action was synchronized with each sentence uttered. The content of the speech was based on samples of caregivers speaking in real-life care environments. To eliminate the bias implicit in choosing a single utterance, six utterances were prepared. The sentences were not dialogs with participants, but rather the robot delivers a short greeting to participants to unify the experimental environment.

### Experimental protocol

For the gentle touch and speech motions of the robot, we referred to the experiment by Toyoshima^25^. In this experiment, the distance of the gentle touch was 15 cm in the middle of the participant’s back, and the robot performs a gentle touch for 10 s in that distance. The gentle touch force was set at 3 N, and the gentle touch speed was 5 cm/s. The speaking speed was 6.5 mora/s (6–7 mora/s), which corresponded to a gentle touch speed of 5 cm/s, and which was based on modeling conducted in preliminary experiments. In the preliminary experiments, we investigated the relationship between the combination of the gentle touch and speech with different parameters and the positive emotions of participants on the basis of positive emotions reports. The temperature of the end-effector was set to close to the participant’s body temperatures. For the speech in the speech alone and touch with speech conditions, the following six sentences were made equally based on the daily nursing speech environment.

1. Hello. How is your health? Do you feel any pain?
2. Hello. Did you sleep well? Have a good day.
3. Hello. Please take care of yourself. It has been chilly these days.
4. Hello. How are you doing? Feel free to tell me anything.
5. Hello. How are you doing? Did you sleep well last night?
6. Hello. Are you cold? Do you feel any pain?

The experiment consisted of a total of 36 trials presented in two blocks of 18 trials, with an equal number (i.e., six trials) of touch alone, speech alone, and touch with speech conditions. The trial order was randomized in terms of conditions and sentences within the block. A short recess was taken after the first block. Before the experiment began, participants engaged in 3 practice trials to gain familiarity with the procedure.

For each trial, a beep sound was presented as a warning signal. After the beep, the robot arm moved to and touched the participant, and paused for 10 s as the baseline period in the touch alone and the touch with speech conditions; the robot arm moved (to create the same sound) but did not touch in the speech alone condition. Then, the stimulus of each of the three conditions was presented for 10 s, after which participants rated their subjective experiences using a questionnaire. The physiological data were continuously recorded for all trials. All participants were asked to look forward toward the dark cloth covering the table. On the table, there were paper questionnaires of the Affect Grid.

### Subjective evaluation

**Figure 4:**
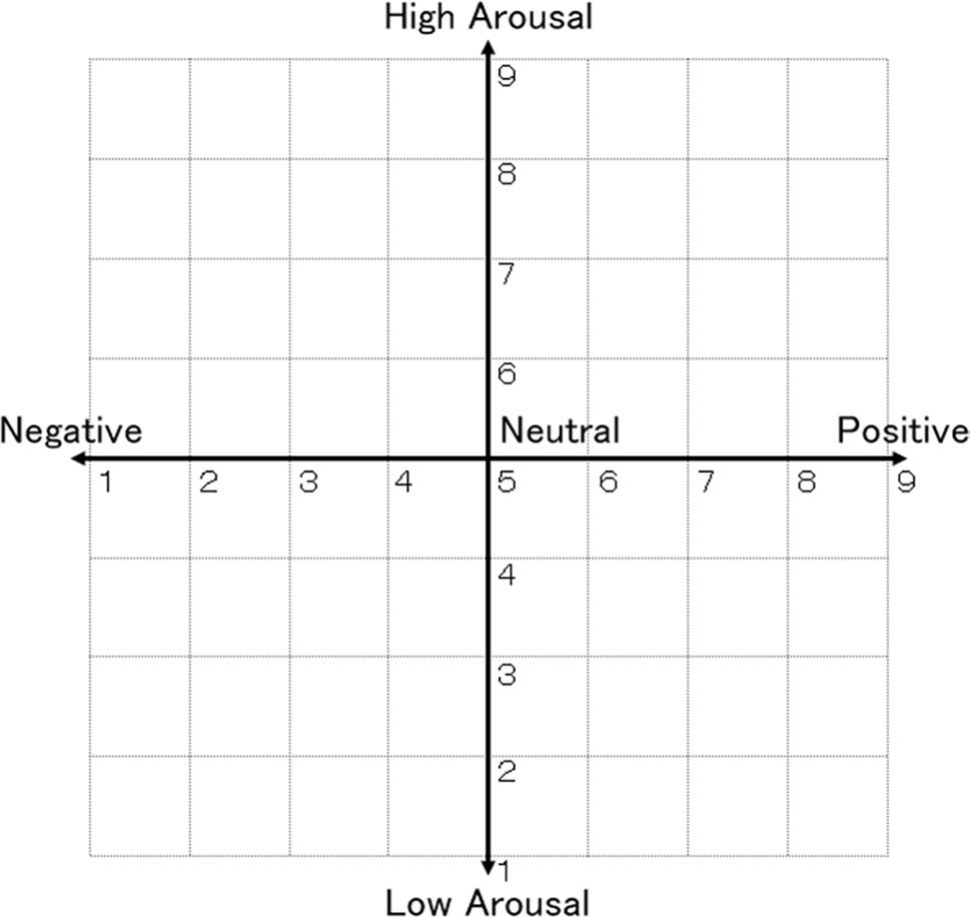
The affect grid for the subjective ratings of valence and arousal.

A questionnaire was used to assess the subjective ratings for the valence, arousal, and human likeliness in response to the robot’s touch. Participants were asked to evaluate their subjective responses in each trial. To examine the valence and arousal effects, the Affect Grid (Fig. 4), an evaluation method for emotion graphically grading emotion was employed^44^. Its center is emotionally neutral, i.e., neither positive nor negative, nor normal. The horizontal axis represents emotional valence, which ranges from negative (left) to positive (right). The vertical axis of the Affect Grid represents the degree of arousal, ranging from high to low, and reflects the intensity of negative or positive emotions. At the beginning of the experiment, participants were instructed to rate their subjective emotional experience in response to the robot action using both axes. To facilitate participants’ understanding of the Affect Grid, further illustrations were provided as follows: “the upper-right corner denotes high positive and high arousal feelings, such as excitement; the lower-right corner represents high positive and low arousal feelings, such as relaxation; the lower-left corner is for high negative and low arousal feelings, such as depression; and the upper-left corner indicates high negative and high arousal feelings, such as stress.” The participant marked the position of the emotion most applicable to each action. For assessing the human likeliness, the participant marked the number on a single Likert-scale item ranging from 1 (not at all humanlike) to 7 (very humanlike), evaluating how the robot action felt humanlike.

We conducted a preliminary analysis for the reliability of these scales. Cronbach’s across trials under each condition was found to be 0.93, 0.91, 0.92, 0.94, 0.89, 0.94, 0.97, 0.95, and 0.96 for valence-touch, valence-speech, valence-touch with speech, arousal-touch, arousal-speech, arousal-touch with speech, human likeliness-touch, human likeliness-speech, and human likeliness-touch with speech, respectively, suggesting the acceptable reliability of the scales.

### Physiological data recording

EMG was recorded using sets of pre-gelled, self-adhesive 0.7 cm Ag/AgCl electrodes with 1.5 cm inter-electrode spacing (Prokidai, Sagara, Japan). The electrodes were placed on the corrugator supercilii and zygomatic major muscles according to guidelines^45,46^ by placing a ground electrode on the forehead. The data were amplified, filtered online (bandpass: 20–400 Hz), and sampled at 1000 Hz using an EMG-025 amplifier (Harada Electronic Industry, Sapporo, Japan), and the PowerLab 16/35 data acquisition system, and LabChart Pro v8.0 software (ADInstruments, Dunedin, New Zealand). Furthermore, unobtrusive video recording was conducted to check for motion artifacts using a digital web camera (HD1080P, Logicool, Tokyo, Japan).

SCL was recorded using pre-gelled, self-adhesive 1.0 cm Ag/AgCl electrodes (Vitrode F, Nihonkoden, Tokyo, Japan). The electrodes were placed on the palmar surface of the medial phalanges of the index and middle fingers of the participants’ left hands according to guidelines^47^. Next, a constant voltage of 0.5 V was applied using a Model 2701 BioDerm skin conductance meter (UFI, Morro Bay, CA, USA). The data were recorded using the same data acquisition system and recording software with the aforementioned EMG, except that there was no online filter.

### Data analysis

#### Preprocessing

One of the authors blindly checked the video data and confirmed that participants did not produce large motion artifacts. EMG data analyses were performed using Psychophysiological Analysis Software 3.3 (Computational Neuroscience Laboratory of the Salk Institute) and in-house programs implemented in MATLAB 2018 (MathWorks, Natick, USA). The data were sampled for 1 s during the pre-touch baseline and for 10 s during the stimulus presentation in each trial. The data for each trial were rectified and baseline-corrected to the average value over the pre-stimulus period, and averaged. Trials with an absolute signal value > 5 *SD* from the mean for each electrode for each participant were rejected as artifacts. The frequency of artifact-contaminated trials was low (0.41 and 0.74% for corrugator supercilii and zygomaticus major, respectively). The values for each stimulus were then standardized within each individual. The same analyses were conducted for the SPL as for the EMG analysis except that the data were not rectified. The frequency of artifact-contaminated trials for SCL was also low (0.25%).

#### Statistical analysis

We performed repeated-measures MANOVAs with condition (touch, speech, touch and speech) and block (first, and second) for within-subjects factors for each subjective rating and physiological signal. The assumption of no multicollinearity was attained (variance inflation factor < 1.78 and < 1.26 for subjective ratings and physiological signals, respectively). For following up the significant effects, univariate ANOVAs and multiple comparisons using the Ryan method (correcting Type I error rate for multiple comparisons in a stepwise manner^49^) were conducted. We reported 95% CIs after being adjusted for multiple comparisons^48^. The results of all of the tests were considered statistically significant at *p* < 0.05.

Because our speech stimuli contained sentences with (i.e., #1, 2, and 4–5) and without questions (i.e., #3), we conducted a preliminary analysis to test this effect. We conducted repeated-measures ANOVAs with condition (speech, touch and speech) and question (with questions, without questions) as within-subject factors for subjective rating and physiological signal data in the conditions with speech. The results showed no significant effect or interaction related to the factor of question (F(1,30) < 1.90, *p* > 0.172, *η*^2^_*p*_ < 0.060). Hence, it was disregarded.
